# Changes in Serum Sphingomyelin After Roux-en-Y Gastric Bypass Surgery Are Related to Diabetes Status

**DOI:** 10.3389/fendo.2018.00172

**Published:** 2018-04-25

**Authors:** Elin Rebecka Carlsson, Josefine Liv Gilling Grundtvig, Sten Madsbad, Mogens Fenger

**Affiliations:** ^1^Department of Clinical Biochemistry, Copenhagen University Hospital Hvidovre, Hvidovre, Denmark; ^2^Department of Endocrinology, Copenhagen University Hospital Hvidovre, Hvidovre, Denmark

**Keywords:** sphingolipids, diabetes, obesity, gastric bypass surgery, metabolic surgery, sphingomyelin, human, clinical research

## Abstract

Metabolic surgery is superior to lifestyle intervention in reducing weight and lowering glycemia and recently suggested as treatment for type 2 diabetes mellitus. Especially Roux-en-Y gastric bypass (RYGB) has been focus for much research, but still the mechanisms of action are only partly elucidated. We suggest that several mechanisms might be mediated by sphingolipids like sphingomyelin. We measured serum sphingomyelin before and up to 2 years after RYGB surgery in 220 patients, divided before surgery in one non-diabetic subgroup and two diabetic subgroups, one of which contained patients obtaining remission of type 2 diabetes after RYGB, while patients in the other still had diabetes after RYGB. Pre- and postoperative sphingomyelin levels were compared within and between groups. Sphingomyelin levels were lower in diabetic patients than in non-diabetic patients before surgery. Following RYGB, mean sphingomyelin concentration fell significantly in the non-diabetic subgroup and the preoperative difference between patients with and without diabetes disappeared. Changes in diabetic subgroups were not significant. Relative to bodyweight, an increase in sphingomyelin was seen in all subgroups, irrespective of diabetes status. We conclude that RYGB has a strong influence on sphingomyelin metabolism, as seen reflected in changed serum levels. Most significantly, no differences between the two diabetic subgroups were detected after surgery, which might suggest that patients in both groups still are in a “diabetic state” using the non-diabetic subgroup as a reference.

## Introduction

Several studies have shown that operations, where a part of the proximal intestine is isolated from ingested food, can treat type 2 diabetes mellitus independent of weight loss (1, 2). The Roux-en-Y gastric bypass (RYGB) procedure has been of special interest since remission of diabetes is often reported shortly after surgery before any significant weight loss, but also because of the major weight loss of about 40 kg the first year after surgery (3). It has been suggested that one of RYGB’s major effects on glucose hemostasis is mediated through an enhanced increase of glucagon-like peptide-1 (GLP-1) during a meal and an improvement of hepatic insulin sensitivity already few days after surgery. Later, following the weight loss also insulin sensitivity in the skeletal muscles is improved (4, 5). Nevertheless, the mechanisms underlying the glycemic effects of RYGB surgery are not fully elucidated and still debated (6).

A link between lipid metabolism and inflammation has been suggested to mediate metabolic disease (7). Key players in this signaling network seem to be sphingolipids (8–10), a large family of lipids with important roles in membrane biology and cellular signaling (11), and there is strong evidence for sphingolipids to be involved in development of insulin resistance (10, 12, 13). Ceramide, is suggested to play a significant role in inducing β-cell apoptosis, as well as in mediating insulin resistance and reducing insulin gene expression (14); sphingosine-1-phosphate, has been shown to improve β-cell function, promote glucose-stimulated insulin release, and protect against β-cell apoptosis (15); and sphingomyelin, which will be described more in details below has been connected to insulin resistance in animal studies (16, 17).

So far, a limited number of studies have connected metabolic surgery and sphingolipids, looking at changes in sphingolipids after mainly RYGB (18–23). Apart from the study by Kayser, they all include a quite small number of patients. The conclusions from these studies are that sphingolipid metabolism changes after RYGB (18–23), that the changes are associated with diabetes remission (22), and that similar changes are not seen after adjustable gastric banding (AGB) (21).

Among the sphingolipids, sphingomyelin is the most abundant (16, 24). It consists of a phosphocholine head group on a body of ceramide (11, 16, 25) and is either synthesized by sphingomyelin synthase from ceramide and phosphocholine or cleaved by sphingomyelinase to ceramide (25–27). Modulation of sphingomyelin content in cellular membranes seem to influence insulin sensitivity in mice models (28), indeed sphingomyelin seem to have important roles in regulating membrane structure, cellular signaling, -migration, and nuclear function (26).

Of the clinical studies mentioned earlier, only the latest three lipidomic studies include measurements of sphingomyelin, specifically (21–23). Graesslers study of five morbidly obese patients, four of which had type 2 diabetes, found no significant change in total plasma sphingomyelin, but 7 out of 22 sphingomyelin species, that differ in their content of fatty acids, were significantly reduced 3 months after RYGB, compared to baseline (23). Arora found a negative correlation between some sphingomyelin species and insulin levels 4 days after RYGB in 16 insulin-resistant subjects, where 14 had type 2 diabetes. Sphingomyelin species were also among the variables that were most discriminatory between time points and at their lowest level 42 days after RYGB (22). In a cohort of 59 obese women, Kayser compared serum lipid profiles at baseline and after either RYGB or AGB. At 1 and 3 months after surgery, respectively, there were 21 and 19 patients left in the RYGB-group. After surgery, 12 patients were left in the AGB-group. In this study, several lipids were differentially affected by the two types of surgery, some of them species of sphingomyelin. Interestingly, monounsaturated sphingomyelin species decreased after RYGB, whereas polyunsaturated sphingomyelin species increased (21).

In this study, we look at sphingomyelin in serum before and after RYGB surgery in a large patient cohort (*n* = 220). We hypothesized that sphingomyelin levels change after RYGB and that changes are related to glucose tolerance and weight loss.

## Research Design and Methods

### Research Population and Biobank

We have previously described the research population of patients treated for obesity between 2009 and 2014 at Hvidovre University Hospital in the Capital Region of Copenhagen (29). From this population, we selected all RYGB-operated patients operated between November 2010 and September 2013, who had delivered a fasting serum blood sample before their operation and, since we were especially interested in early changes after RYGB, also had delivered a fasting sample within 4 months after their operation. We then included all fasting serum blood samples from the same patients up to 2 years after surgery, ending up with a total of 835 samples from 220 patients. Of these samples, 220 were from prior to surgery, and 220, 158, 148, and 89 were from 3, 6, 12, and 24 months after surgery, respectively. Serum samples had all been frozen shortly after sampling at −80°C and stored between 6 months and 5 years at the time for our sphingomyelin analysis. Clinical characteristics as weight and BMI, systolic and diastolic blood pressure were for a majority of the patients registered in a database at several time points during the course of treatment. We also had access to biochemical laboratory data from 2009 and onward. The surgical technique and methods for biochemical analyses are described in the above mentioned paper (29). This study was performed in accordance with the Helsinki Declaration and was approved by the Scientific Ethics Committee of the Capital Region, Denmark, protocol number HD2009-78, extended with the protocol number H-6-2014-029, and by the Danish Data Protection Agency. Informed consent was obtained in writing from all the participants in this study.

### Sphingomyelin Assay Principle

The samples were analyzed in double with an in-house sphingomyelin assay, all samples from the same patient in sequence at the same time of analyzing.

The method was modified from other similar, previously described methods (30, 31), determining the concentration of sphingomyelin by measuring free choline in a 10-µL serum sample after incubation with sphingomyelinase (0.05 U), alkaline phosphatase (5 U), choline oxidase (0.35 U), and peroxidase (15 U) in 100 µL of a buffer of 0.05 M Trishydrochlorid and 0.66 mM Calcium Chloride buffer (pH 8) with 0.05% Triton X-100, added 2 mM DAOS [*N*-ethyl-*N*-(2-hydroxy-3-sulfopropyl)-3,5-dimethox-yaniline] and 0.72 mM 4-aminoantipyrin. Instead of using a standard of sphingomyelin-extract from a biological source, we chose to use a standard solution of choline chloride (10,000 µmol/L diluted with buffer to concentrations between 250 and 750 µmol/L) and used sphingomyelin (diluted in 2% triton X-100 in ethanol) for assay level control. Reagents were choline oxidase (037-14401) from Wako Chemicals GmbH and (C4405) from Sigma-Aldrich; sphingomyelinase (S8633), alkaline phosphatase (P6774), peroxidase (P6782), 4-aminoantipyrine (A4382), DAOS (E8381), sphingomyelin (S0756), choline chloride (C7017), and Triton X-100 from Sigma-Aldrich Denmark A/S; sphingomyelin (860061 C) from Avanti Polar Lipids, Inc.; Ultrapure™, Trishydrochlorid, and ethanol from Merck & Co., Inc.; and calcium chloride from Invitrogen (Thermo Fisher Scientific).

End point absorbances at 595 nm were read spectrophotometrically after 60 min in 37°C by SpectraMax i3x from Molecular Devices on a standard 96-well microplate. Results were calculated with the software SoftMax Pro 6.4 and could be reproduced in double within the same run with a SD 5.6 µmol/L and a CV at 1.3%. Intermediary precision SD was 7.9 µmol/L and the CV 1.6% at the level of 495 µmol/L over five independent time points of analysis. Linearity was documented by serial dilution of a sample with high concentration from 82 to 582 µmol/L and recovery when adding sphingomyelin of known concentration to a sample with an endogenous sphingomyelin concentration at 365 µmol/L was between 101 and 106%. We also checked for possible interference by phosphatidylcholine, by adding known concentrations of phosphatidylcholine to a sample. This did not raise the measured concentration of sphingomyelin.

Apart from serum, heparin plasma can also be used in this assay, but not plasma treated with EDTA or citrate. No lipid extraction is necessary.

As endogenous free choline and possibly free hydrogen peroxide can interfere, we repeated the analysis with a lower set of standards and without sphingomyelinase and alkaline phosphatase and then adjusted the result from the sphingomyelin measurement. This part of the assay had SD 1.7 µmol/L and CV of 5.3% for within run double determinations and the intermediary precision SD 2.2 µmol/L and CV of 5.8%.

### Definition of Remission and Subdivision Into Subgroups According to Diabetes Status

Based on the cutoff limits in WHO’s diagnostic criteria for diabetes mellitus (DM), patients were assumed to have DM only if available lab data could confirm measure of HbA1c at 6.5% (48 mmol/mol) or above, fasting glucose at 7 mmol/L or above, or glucose at 2-h of a glucose tolerance test at 11.1 mmol/L or above. We used the criteria for remission suggested by American Diabetes Association (ADA) in 2009 (32), but without distinguishing between partial, complete, or prolonged remission. In practice, a patient was said to be in remission after RYGB if HbA1c decreased to below 6.5% (48 mmol/mol) without any antidiabetic medication and then stayed low for as long as there were available clinical data, varying from 2 years to a maximum of 5 years.

We divided the 220 patients into five different subgroups: NDM, a non-DM group (*n* = 151); DMH-NDM, a group with DM and hyperglycemia in remission after RYGB (*n* = 34); DMH-DMH, a group with DM and hyperglycemia not in remission after RYGB (*n* = 20); DMT-NDM, a heterogeneous group with possible DM and in treatment with diet or antidiabetic medication before RYGB, but with no confirmed hyperglycemia in lab data and off antidiabetic treatment after RYGB (*n* = 14); DMH-DMT, one patient with DM and hyperglycemia before RYGB in biochemical remission but still on antidiabetic treatment after RYGB (*n* = 1). Only the first three subgroups were compared in statistical analyses and further discussed in this paper.

### Reference Population

To establish a reference interval, we picked 238 samples from a biobank with samples from a healthy normal weight population. Samples had all been stored for about 15 years at −80°C, thawed and refrozen a few but equal times.

### Adjustment of HbA1c Levels Before January 2013

Scandinavian laboratories that are using high pressure liquid chromatographic (HPLC) methods for determining HbA1c were until January 2013 measuring higher levels compared to the rest of Europe (33). This systematical bias was eventually found to be due to the used calibrator, which was then substituted with another, resulting in lower levels than previously, but in agreement with international reference levels. When comparing HbA1c levels measured with HPLC that spread over time on both sides of this calibrator change, we have followed recommendations and adjusted levels analyzed before January 2013 with −2.7 mmol/mol.

### Statistical Analysis

Overall, data are expressed as mean with a 95% confidence interval or SD. Correlations are shown as Pearson or Spearman correlation coefficients *r* or *r*_s_, with the degree of freedom and *p*-value. We have used IBM SPSS version 22 for all analysis.

As most of the parameters are normally distributed and with homogeneity of variances, we have used parametric tests like the one-way ANOVA and Tukey *post hoc* test for our multiple comparisons between groups, the independent t-test for comparing only two subgroups and a paired *t*-test for comparing the same group of patients before and after RYGB. In situations where Levene’s test of equality of variances was significant, we performed the one-way ANOVA with a Welch–Satterthwaite correction followed by a Games–Howell *post hoc* test. If there were outliers that were considered extreme when assessed by inspection of a boxplot (and this was not due to any analytical error) we performed statistical analysis both with and without the outliers in the data material. Generally, removing the outliers did not change but rather increased significance. In order not to overestimate significance, we have, therefore, chosen to report the more conservative *p*-values from analysis with all possible outliers in the material. Only a few of our parameters, like for instance HbA1c and triglycerides did not have a normal distribution. Where this was the case, and differences between subgroups were statistically significant using the normal one-way ANOVA, we confirmed the statistical significance with a Kruskal–Wallis *H*-test. Linear relationships between lipids were determined by Spearman’s correlation. Linear relationships between sphingomyelin and weight were determined by the Pearson correlation. *p*-Values lower than 0.05 were considered significant.

As weight and BMI in this population were not stationary after RYGB, and because this change in mass and volume possibly could have different influence on different parameters, we chose to standardize lipid concentration to bodyweight or BMI in some statistical analyses.

## Results

Of our 220 patients, 151 (NDM) did not have DM and 54 had DM that we were able to confirm in biochemical laboratory data. Of the patients with diabetes, 34 (DMH-NDM) were able to control glucose levels at HbA1c < 6.5% (48 mmol/mol) after RYGB without antidiabetic medication, but 20 (DMH-DMH) still had high HbA1c values after RYGB. As described in the Section “Research Design and Methods,” 15 patients were not in any of these three groups.

The serum samples were drawn 4.54 (4.10–4.99) months prior to surgery, followed up by serum samples at 3.07 (3.03–3.12), 6.44 (6.26–6.63), 12.39 (12.12–12.66), and 24.23 (23.73–24.74) months after surgery. Total-, HDL-, LDL- and VLDL-cholesterol were analyzed 5.86 (6.42–5.31) months prior to surgery and 2.60 (2.54–2.67), 6.50 (6.36–6.64), 12.04 (11.83–12.25), and 24.17 (23.91–24.43) months after surgery. HbA1c were analyzed 4.38 (3.87–4.89) months prior to surgery and 3.09 (3.04–3.14), 6.52 (6.38–6.66), 12.81 (12.51–13.11), and 26.33 (25.47–27.19) months after surgery.

Patients in the two diabetic subgroups were older and had lower levels of total- and LDL-cholesterol, compared to the NDM subgroup (Table 1). The patient population as a whole was comparable to the reference group on many parameters apart from weight and BMI, but there were differences in lipid concentrations. Noticeably, the reference group HbA1c levels were significantly higher than those in the NDM patient group, indicating that there have been some individuals with undiagnosed diabetes in the reference group. Sphingomyelin levels were similar in patient- and reference population, but significantly lower for diabetic than non-diabetic patients before surgery (*p* < 4.3 e^−8^). Weight and BMI distribution, as well as postoperative weight loss and changes in BMI, were at all time points similar in all subgroups (Table S1 in Supplementary Material). As described previously, female and male patients were comparable before surgery as regarding to age, weight, BMI, blood pressure, HbA1c, and plasma cholesterol (29). HbA1c concentrations, as shown in Table S1 in Supplementary Material, decreased after surgery in all subgroups. Noteworthy, in the DMH-NDM group, mean HbA1c at all time points after surgery were within the normal range, not just below the diabetic threshold. In the DMH-DMH group, mean HbA1c decreased to sub-diabetic, still hyperglycemic levels at 6 and 12 months after surgery, but returned to diabetic levels at 24 months. Mean sphingomyelin concentration in the whole group was 413.1 (400.7–425.5) μmol/L before surgery. There was a significant difference between genders, as the concentration was higher in female than in male patients, 434.3 (419.8–448.9) μmol/L compared to 367.6 (347.8–387.4) μmol/L (*p* < 3.9 e^−7^). The gender difference was also clear in the reference population, females 460.8 (443.8–477.8) μmol/L and males 371.1 (351.1–391.1) μmol/L (*p* < 9.9 e^−11^).

**Table 1:** Preoperative clinical characteristics and sphingomyelin (SM) concentration for all patients and patients grouped according to diabetes status, alongside a normal weight reference population.

	All patients (*n* = 220)^a^	NDM (*n* = 151)	DMH-NDM (*n* = 34)	DMH-DMH (*n* = 20)	ANOVA *p*-value^†^	Reference (*n* = 238)	*t*-Test *p*-value^‡^
				
	Mean (SD)	Mean (SD)	Mean (SD)	Mean (SD)		Mean (SD)	
Age (years)	44.6 (9.5)	42.1 (9.0)	50.5 (8.1)*	51.5 (7.4)*	7 e^−9^	45.5 (8.9)*	2 e^−4^
Gender (f/m)	150/70	113/38	18/16	9/11	ND	121/119	ND
Height (cm)	171.8 (9.6)	171.0 (9.8)	174.5 (8.0)	171.2 (11.4)	0.167	171.1 (9.3)	0.946
Weight (kg)	125.2 (21.6)	126.3 (22.4)	126.4 (20.6)	117.6 (19.7)	0.244	79.3 (15.4)*	2 e^−61^
BMI (kg/m^2^)	42.3 (5.8)	43.1 (5.9)	41.4 (5.4)	40.0 (3.6)*	0.006	27.0 (4.5)*	2 e^−81^
Systolic BP (mmHg)	128 (14.7)	126.8 (14.9)	131.2 (12.6)	128.2 (14.7)	0.285	132.1 (19.2)*	0.004
Diastolic BP (mmHg)	82.2 (10.1)	82.0 (11.0)	81.4 (6.8)	82.4 (10.1)	0.93	82.3 (16.7)	0.829
HbA1c (mmol/mol)	39.0 (9.7)	34.5 (3.8)	48.8 (11.7)*	55.2 (10.0)*	4 e^−12^	40.6 (5.8)*	6 e^−26^
HbA1c (%)	5.7 (0.89)	5.3 (0.35)	6.6 (1.07)	6.9 (0.91)	ND	5.9 (0.53)	ND
Cholesterol (mmol/L)							
Total-	4.74 (1.04)	4.98 (0.96)	4.22 (1.11)*	4.24 (1.17)*	4 e^−5^	5.52 (1.08)*	8 e^−7^
HDL-	1.15 (0.30)	1.19 (0.29)	1.06 (0.36)	1.09 (0.35)	0.038	1.35 (0.32)*	2 e^−6^
LDL-	2.88 (0.95)	3.12 (0.85)	(*n* = 32) 2.36 (0.97)*	(*n* = 18) 2.27 (1.13)*	1 e^−6^	(*n* = 68) 3.69 (0.97)*	2 e^−5^
VLDL-	0.71 (0.32)	0.67 (0.30)	(*n* = 32) 0.75 (0.30)	(*n* = 18) 0.89 (0.47)	0.086	(*n* = 68) 0.58 (0.25)*	0.033
Triglycerides (mmol/L)	1.67 (1.09)	1.50 (0.74)	2.11 (1.95)	2.13 (1.26)	0.038	1.27 (0.83)*	0.006
SM (μmol/L)	413.1 (93.2)	437.2 (84.0)	360.3 (101.2)*	343.2 (84.0)*	4.5 e^−8^	416.70 (111.4)^#^	0.04
*Female patients*	434.3 (90.3)	449.1 (83.7)	384.9 (105.6)*	381.6 (105.0)	0.003	460.8 (94.5)	0.32
*Male patients*	367.6 (83.0)	401.7 (75.3)	332.6 (91.4)*	311.7 (46.6)*	0.001	371.1 (109.4)	0.11

*Data are reported as mean (SD). If the number of patients with available clinical data was less than 95% of the total of patients in the group, the actual number is specified. Age is on the day of surgery. Clinical data represent the closest available before surgery. NDM, patients without diabetes mellitus (DM); DMH-NDM, patients with DM in remission after Roux-en-Y gastric bypass surgery (RYGB); DMH-DMH, patients with DM not in remission after RYGB; Reference, a population of healthy individuals with normal weight; ND, not determined; SM, sphingomyelin concentrations are shown for all, followed by concentrations for female and male patients, respectively*.

*^a^All patients also include 15 patients who belong to other subgroups than the three showed in table*.

*^†^p-Value from ANOVA comparing the three patient subgroup means*.

*^‡^p-Value from independent samples t-test comparing the reference group mean to the mean in the patient subgroup NDM*.

*Indicates significant difference (*p* < 0.05) when compared to the NDM group. No significant difference was found between the two diabetes subgroups. The two diabetes subgroups were not compared to the reference group.

*^#^SM concentration in serum is higher in women than in men (*p* = 3.9 e^−7^). This explains the low *p*-value in the comparison of reference population and NDM group, as the gender distribution is different in the two groups. *Post hoc p*-values from Tukey and Games–Howell are not shown in table*.

**Figure 1:**
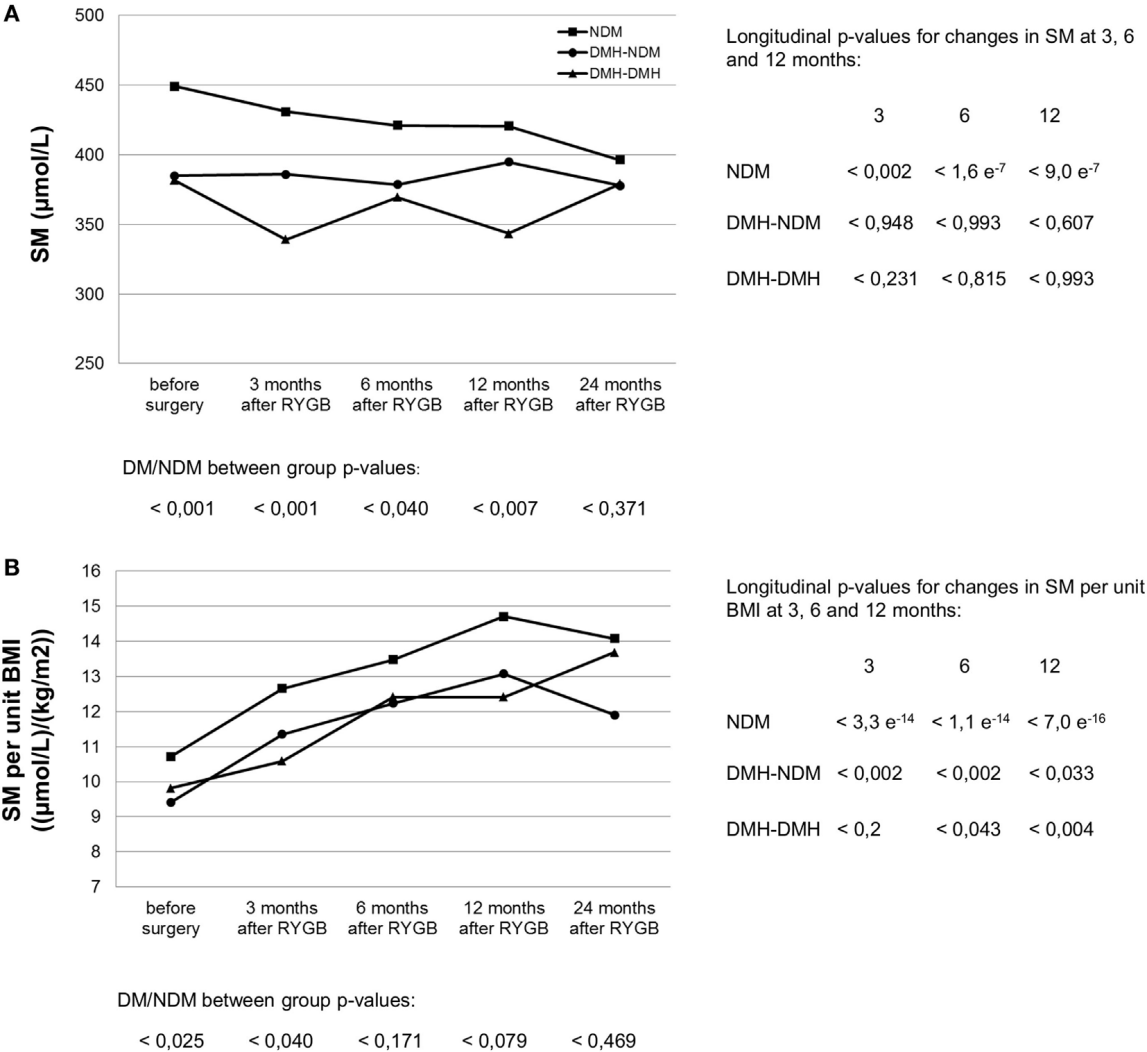
Serum sphingomyelin (SM) concentration **(A)** and its relation to body mass index **(B)** before and after RYGB surgery in non-diabetic and diabetic patient subgroups. NDM, patients without diabetes mellitus (DM); DMH-NDM, patients with DM in remission after RYGB; DMH-DMH, patients with DM not in remission after RYGB. Examples are from the female subpopulation. Male subpopulation showed similar trend, as seen in Table S2 in Supplementary Material. The curves for SM per kilogram bodyweight were similar to the ones per unit BMI shown in **(B)**. *p*-Values from independent samples *t*-tests comparing patients with diabetes (DM) with patients without diabetes (NDM) at each time point are shown at the bottom of each figure. No statistical significance was found between the two diabetic subgroups. Longitudinal *p*-values from paired *t*-tests comparing postoperative SM at 3, 6, and 12 months after surgery with corresponding values before surgery are shown in columns to the right of the graph.

**Figure 2:**
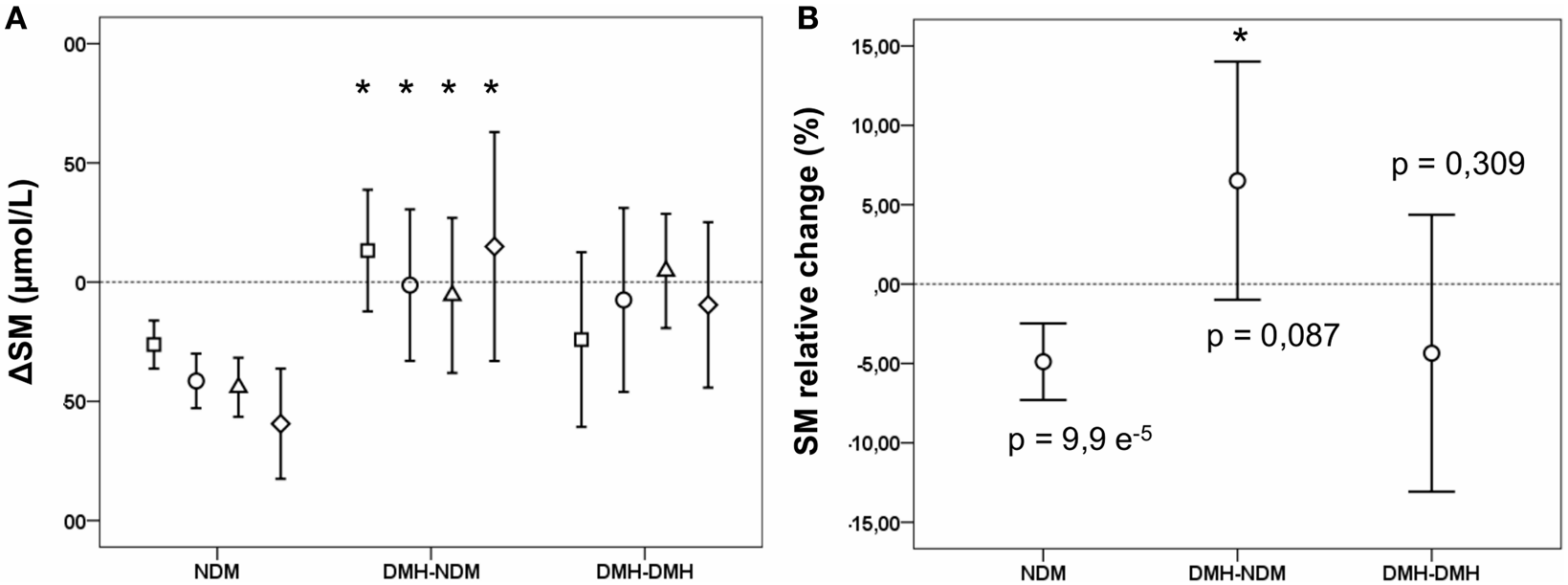
Changes in sphingomyelin (SM) concentration after Roux-en-y gastric bypass (RYGB) surgery in non-diabetic and diabetic subgroups. Figures show mean and a 95% confidence interval of the mean for differences (Δ SM) between SM concentrations before surgery and at 3 (squares), 6 (circles), 12 (triangles), and 24 months (diamonds) after RYGB, respectively **(A)**; and the relative change in SM at 3 months after surgery in percent **(B)**. NDM, patients without diabetes mellitus (DM); DMH-NDM, patients with DM in remission after RYGB; DMH-DMH, patients with DM not in remission after RYGB. Shown *p*-values are from a one-sample *t*-test, comparing the change with 0. Significantly different values when compared to corresponding value in the NDM subgroup is marked with a * (*p* < 0.05).

Following RYGB, sphingomyelin mean concentration for the whole patient population was significantly reduced to 394.0 (382.2–405.8) at 3 months after surgery (*p* < 2.6 e^−5^), and stayed at the new level during follow-up (Figure 1A; Table S2 in Supplementary Material). This decrease was explained by a decrease in the NDM group, whereas changes in sphingomyelin levels in the two diabetic subgroups were small and not statistically significant. During follow-up, the difference observed before surgery between diabetic and non-diabetic subgroups disappeared, for male patients this occurred already 6 months after surgery, while the difference for female patients was still significant at 12, but not at 24 months after surgery. Although not significantly different from 0, delta (Δ) sphingomyelin values showed a positive trend in the DMH-NDM subgroup (Figure 2A), with mean Δ-values significantly different from mean Δ-values in the NDM subgroup (*p* < 0.007). Also the percentage relative changes (Figure 2B), clearly shows that the significant decrease in sphingomyelin in the non-diabetic subgroup was not observed in the diabetic subgroups. The differences between patients with and without diabetes were independent of gender. There was no linear relationship between sphingomyelin and weight before surgery, and no association between change in sphingomyelin and weight loss. There was although a significant negative correlation between weight and sphingomyelin at 12 and 24 months after surgery [*r*(90) = −0.284, *p* < 0.006] and [*r*(24) = −0.414, *p* < 0.035], respectively. In all patients, weight at all time points after surgery correlated strongly and with high significance with weight before surgery. Also, post- and preoperative sphingomyelin concentrations were strongly correlated.

**Figure 3:**
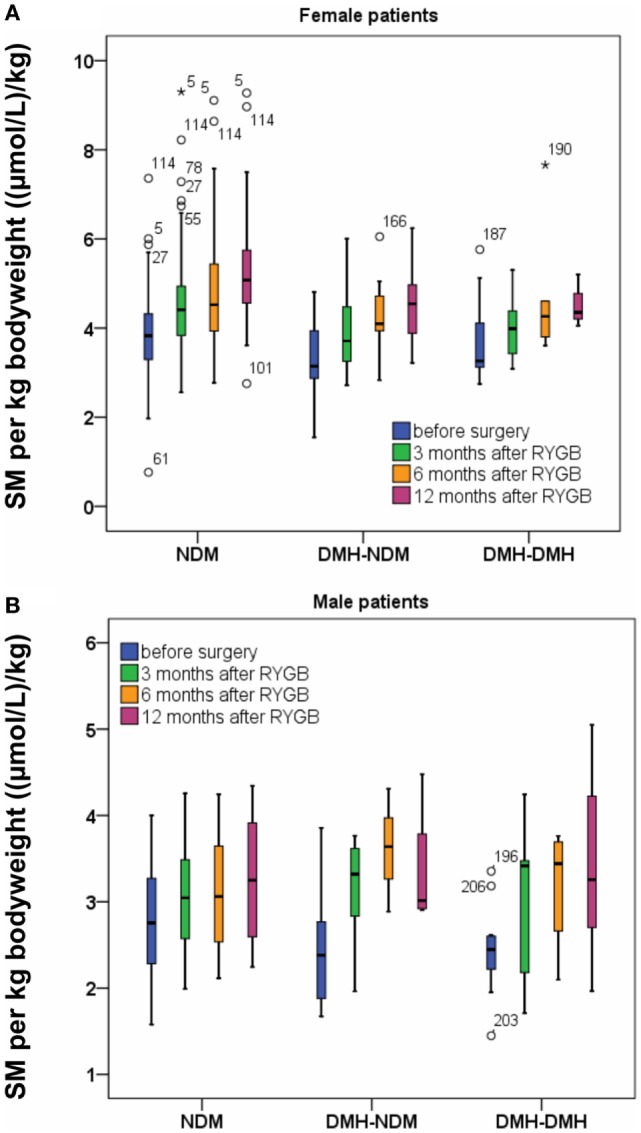
Sphingomyelin (SM) per kilogram bodyweight before and after Roux-en-Y gastric bypass (RYGB) surgery for female patients **(A)** and male patients **(B)**. Figure show boxes with median, 25 and 75% centiles before surgery and at 3, 6, and 12 months after RYGB.

Standardized to body weight or BMI, sphingomyelin concentration rose significantly after RYGB per kilogram bodyweight (Figure 3) or unit BMI (Figure 1B; Table S2 in Supplementary Material) in all patient subgroups, irrespective of diabetes status. In percent, relative postoperative increase in sphingomyelin concentration per kilogram bodyweight was largest in the two diabetic subgroups (Table 2) and significant for male patients. In relation to cholesterol, the ratio between sphingomyelin and total cholesterol concentration in moles per liter increased from 0.088 (0.086–0.090), before surgery, to 0.101 (0.099–0.103) 3 months after RYGB (*p* = 2.6 e^−32^). During the next 2 years of follow-up, the ratio gradually decreased to the same level as before surgery.

**Table 2:** Relative percentage increases in sphingomyelin concentration per kg bodyweight at 3, 6, and 12 months after RYGB surgery.

		NDM		DMH-NDM		DMH-DMH		*p*-Value
					
		*n*	Mean (95% CI)	*n*	Mean (95% CI)	*n*	Mean (95% CI)	
								
Months after RYGB			%		%		%	
3	All	116	16.0 (13.0–19.1)	26	25.6 (15.2–36.1)	14	21.1 (9.3–32.9)	0.052
	Females	89	17.5 (13.9–21.2)	16	21.5 (7.1–35.9)	5	8.5 (-1.1–31.7)^a^	0.398
	Males	27	11.2 (6.2–16.1)	10	32.3 (15.3–49.2)	9	28.2 (12.3–44.0)	0.002
6	All	84	19.6 (15.8–23.4)	15	38.5 (16.8–60.2)	11	31.1 (15.9–46.3)	0.091
	Females	64	21.9 (17.7–26.0)	12	36.3 (9.9–62.8)	5	26.3 (6.9–49.5)^a^	0.498
	Males	20	12.5 (3.6–21.4)	3	47.2 (17.7–81.8)^a^	6	35.1 (7.5–75)^a^	0.016
12	All	66	29.0 (23.6–34.4)	10	44.0 (20.9–67.2)	11	41.4 (26.7–56.2)	0.068
	Females	50	32.8 (26.8–38.9)	6	31.3 (4.1–58.5)	3	28.5 (24.3–32.7)	0.418
	Males	16	17.1 (6.2–28.0)	4	63.2 (18.3–96.0)^a^	8	46.3 (26.0–66.6)	0.002

*Data are shown in percent (%) as mean with a 95% CI, except in ^a^, were CI where larger than minimum and maximum values, which are reported instead. NDM, patients without diabetes mellitus (DM); DMH-NDM, patients with DM in remission after RYGB; DMH-DMH, patients with DM not in remission after RYGB; *n*, number of patients; RYGB, Roux-en-Y gastric bypass*.

**Figure 4:**
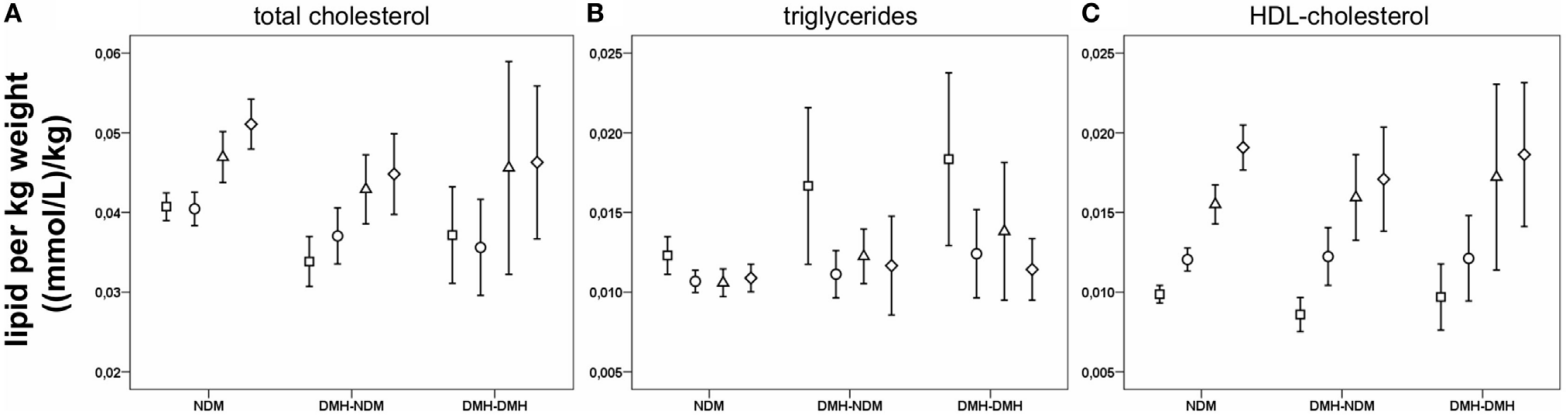
Lipids per kilogram bodyweight before and after Roux-en-Y gastric bypass (RYGB) surgery. Figure show mean and a 95% confidence interval of the mean before surgery and at 3, 6, and 12 months after RYGB for the whole patient population for total cholesterol **(A)**, triglycerides **(B)** and HDL-cholesterol **(C)**.

Lipid concentrations at all time points are shown in Table S1 in Supplementary Material. Total cholesterol after RYGB was initially reduced followed by a slight increase during the rest of follow-up, probably due to the increase in HDL-cholesterol after 6 months. All subgroups follow the same pattern of changes in total cholesterol and HDL-cholesterol. LDL-cholesterol showed a significant reduction at 3 months after surgery, but for the diabetic subgroups, the change was no longer significant after 6 months. Triglycerides, was reduced in all subgroups after RYGB, with a continuous downward trend during follow-up. VLDL-cholesterol decreased significantly after RYGB in all subgroups and stayed low. Figures 4A–C show changes in cholesterol, triglyceride, and HDL-cholesterol concentrations standardized to bodyweight. For LDL- and VLDL-cholesterol standardized to bodyweight, changes after RYGB were smaller or less significant.

Sphingomyelin levels correlated positively with total-, HDL-, and LDL-cholesterol before and after surgery (Table S3 in Supplementary Material). The correlations were strongest between sphingomyelin and total cholesterol [*r*_s_(218) = 0.801, *p* < 1.5 e^−50^] as well as between sphingomyelin and LDL-cholesterol [*r*_s_(218) = 0.731, *p* < 4.9 e^−38^] at 3 months after surgery. Twelve months after surgery, the correlation between sphingomyelin and LDL-cholesterol was less strong than before surgery [*r*_s_(136) = 0.527, *p* < 3.0 e^−11^]. The correlation between sphingomyelin and HDL-cholesterol at 3 months after surgery was moderate [*r*_s_(218) = 0.350, *p* < 1.1 e^−7^], but was stronger 12 months after surgery [*r*_s_(136) = 0.518, *p* < 7.8 e^−11^]. Sphingomyelin did not correlate with VLDL or triglycerides.

## Discussion

Previous studies have reported a decreased serum or plasma sphingomyelin concentration after weight loss in humans (34, 35). We could not confirm any linear relationship between sphingomyelin and body weight in all our patients, except for weak correlations at 12 and 24 months after surgery, where a higher body weight was associated to a lower sphingomyelin concentration. We found a decrease in level of sphingomyelin after weight loss following RYGB in the non-diabetic subgroup, but not in the diabetic subgroups. Also, patients with diabetes had a significantly lower sphingomyelin concentration before surgery than patients without diabetes.

The source of serum sphingomyelin is not known. Dietary sphingomyelin does not seem to be a source of circulating sphingomyelin as it is slowly and incompletely digested by intestinal alkaline sphingomyelinase (alkSMase) and neutral ceramidase to sphingosine (36). Intact sphingolipids or ceramides are not absorbed, but sphingosine is to a large extent (11, 24, 36). Considering that alkSMase is active primarily in the jejunum and depends on bile in order to be active in the gut lumen (25), RYGB surgery bypassing duodenum and the pancreatic duct may entirely change the gut metabolism of sphingolipids. The increased load of sphingomyelin to the distal intestine and colon may change its absorbance kinetics, of which we however have no information about. The liver seems to be the major source of serum lipids (37). Human liver contains higher concentrations of ceramides and saturated fatty acids than subcutaneous and visceral fat, but it is not known if this extends to high concentrations of sphingomyelin generated by sphingomyelin synthesis in the liver.

Sphingomyelin is together with cholesterol a major constituent of cellular membranes (17), influencing membrane fluidity (38), and the proportion of sphingomyelin in cellular membrane lipid rafts affect receptor signaling, likely by regulating receptor accumulation and dimerization (39). By preventing insulin receptor dimerization, lipid raft alterations are suggested to play a role in development of insulin resistance (28), and DM may arise as a consequence of an unbalanced membrane sphingolipid composition (40). In addition, degradation of membrane sphingomyelin to ceramide increases ceramide concentration in lipid rafts, which in the pancreas promotes beta cell apoptosis (14). It is not clear, if sphingomyelin serum levels reflect the alterations in the plasma membrane seen in DM.

The levels of serum sphingomyelin were significantly higher in patients without diabetes compared to patients with diabetes before surgery, while no difference was seen between the two diabetic subgroups. For female patients, the difference between the diabetic subgroups on one side and the non-diabetic subgroup on the other still persisted at 12, but not 24 months after RYGB. For male patients, the difference was no longer significant at 3 months after RYGB. After RYGB, sphingomyelin levels only decreased in the patients without diabetes. This suggests that sphingomyelin is physiologically related to the metabolic state of the patients and that sphingomyelin reflects the glucose tolerance state. The causal relationship is, however, far from clear. Also, considering the specificity of our assay that measures total sphingomyelin, it is possible, that the constant sphingomyelin concentration in diabetic subgroups may cover over combined increases and decreases in individual sphingomyelin species.

Intriguingly, although absolute sphingomyelin levels were stationary after RYGB in diabetic subgroups, sphingomyelin levels per BMI unit or kg bodyweight differed marginally between patients with and without diabetes and increased almost in parallel for all patient subgroups, including the diabetic. This suggests that the changed anatomy after RYGB results in a general alteration of sphingolipid metabolism which is independent of diabetes. In contrast, serum levels of sphingomyelin seem to be indigenously related to the diabetic state. Differences between genders might be explained by differences in distribution of body mass and fluid compartments.

Sphingolipid metabolism is likely to have an important role in regulating intestinal absorption of other lipids, like cholesterol (25). The strong correlations that we saw between serum sphingomyelin and plasma total- and LDL-cholesterol indicate that their metabolisms or ways of transport also are connected in serum/plasma. Sphingomyelin is known to be the most abundant sphingolipid in circulating LDL (16). Correlations between LDL-cholesterol and sphingomyelin were strong before surgery and for a short time after, but diminished later after RYGB. This pattern was also seen for correlation to HDL-cholesterol, but in the reverse direction, which might be a reflection of an altered lipoprotein particle composition, including transfer of sphingolipids between LDL- and HDL-particles and their receptors. Total- and LDL-cholesterol were significantly lower in the two diabetic subgroups compared to the patients without diabetes. The likely reason for this was a more frequent treatment with statins in patients with diabetes, as 59% of diabetic patients compared to only 9% of non-diabetic patients were prescribed cholesterol lowering drugs before surgery. After RYGB, LDL-cholesterol stayed lower in the group with persistent diabetes. This also was a likely effect of differences in statin treatment between the subgroups. Statins do not to interfere with sphingomyelin synthesis (41).

Patients in the two diabetic subgroups were 8–9 years older than patients in the non-diabetic group. As both elevated triglycerides and increased insulin resistance are known symptoms of the metabolic syndrome associated with obesity (42), we believe that some of the differences between the older diabetic subgroups and younger non-diabetic subgroup are expressions for the natural steps in development of a metabolic syndrome. We saw remarkable changes in triglycerides after RYGB in all subgroups. Knowing that triglycerides are only one chemical reaction away from diacylglycerol (DAG), the source of glycerophospholipids, likely to contribute to metabolic disease (16, 43) and that DAG and sphingomyelin metabolisms are connected (43), it seems plausible, that mechanisms affected by RYGB improving glucose hemostasis are upstream to both DAG and triglycerides, perhaps regulators of sphingolipid synthesis.

Our aim was to examine which effect RYGB had on serum sphingomyelin concentration in a population where the patient acts as its own clinical control before and after surgery. An obvious problem with this is that several factors cannot be considered stationary in a patient that at the same time undergoes a large weight reduction. At the time for the first postoperative blood sample 3 months after surgery, there had already been a substantial mean weight loss of 18% or 22 kg, which for a majority of patients was more than 50% of the total weight loss after RYGB. The weight loss makes it difficult to draw conclusions on whether the changes in sphingomyelin and other lipids are reflections of a change in metabolism after RYGB or merely products of changes in body mass. To address this to some point, we chose to look at lipid concentrations adjusted to body weight and BMI, or relative to total cholesterol.

A limitation is that some pre-surgery serum samples were drawn months prior to RYGB, where the ideal would have been on the morning of surgery. Some changes in sphingomyelin concentration could, therefore, in theory be occurring already pre-surgically. There was, however, no significant difference between patients with or without diabetes in mean time between blood sampling and operation, so a pre-surgical explanation would not sufficiently cover the observed difference between these patient groups.

The fact that the absolute values of sphingomyelin did not change in patients with diabetes, regardless of normalization of HbA1c, corroborates the notion that the diabetes state persists in all diabetic patients and that obesity and diabetes are separate, although intertwined conditions (29). Importantly, the natural reference group is the group of patients with no diabetes prior to surgery, and any changes in variables after RYGB should be compared to this reference group after RYGB. This is the case for fasting blood sugar (29) and sphingomyelin levels. HbA1C, which is a pivotal diagnostic variable, might also be in need of adjustment when used in a RYGB-operated population, as we see that HbA1c levels decrease after surgery in all subgroups, but although HbA1c is normalized in one of the diabetic subgroups, the difference in HbA1c between these patients and the patients in the non-diabetic subgroup still persists after RYGB (*p*-values <7.8 e^−5^, <0.001, <3.3 e^−4^, <2.2 e^−4^ at 3, 6, 12, and 24 months after surgery, respectively.) The gap is even bigger, of course to the patients in the group with persistently high HbA1c. These non-responders to RYGB can perhaps, as suggested by Rubino et al., come to be classified as one or several new subtypes of type 2 diabetes (44). In order not to overestimate diabetes remission rate after RYGB, we chose a relatively defensive definition of remission and a rough division in two main diabetic groups. If we were to use ADA’s full remission criteria (32), some of the patients in the group with persistent hyperglycemia might in fact be partial responders, underlining the heterogeneity of this group. Complete responders, on the other hand, make up the vast majority of patients in the group with normalized HbA1c levels, making this group more homogenous.

Most research in the field of sphingolipid metabolism has been conducted on non-human beings, particularly mice. Caution should be shown when conjecturing from mice to humans, as the composition of lipoprotein particles differ between the two (45) and this may have an implication on the effects of sphingolipids. In addition, we only have scarce knowledge of the composition of sphingomyelin and of sphingolipids in general (46). Future studies on these issues including the genetic structure of the vast lipid metabolic and regulatory network are needed.

## Ethics Statement

This study was performed in accordance with the Helsinki Declaration and was approved by the Scientific Ethics Committee of the Capital Region, Denmark, protocol number HD2009-78, extended with the protocol number H-6-2014-029, and by the Danish Data Protection Agency.

## Author Contributions

EC and MF designed the study, developed the sphingomyelin assay, interpreted, and discussed the results. EC analyzed all samples, performed data analysis, and drafted the manuscript. JG curated and validated the clinical data files. SM contributed to study design and interpretation of results. All authors reviewed, edited, and approved the final manuscript.

## Conflict of Interest Statement

The authors declare that the research was conducted in the absence of any commercial or financial relationships that could be construed as a potential conflict of interest.
