# Supplementary material for: Changes in Serum Sphingomyelin After Roux-en-Y Gastric Bypass Surgery Are Related to Diabetes Status
Source: Front Endocrinol (Lausanne). 2018 Apr 25;9:172. doi: 10.3389/fendo.2018.00172 (PMC5996901; doi:10.3389/fendo.2018.00172)
Supplement: Supplementary file 1 [file table_1.PDF]

Supplementary Table S1. Preoperative clinical characteristics and surgical outcomes off all patients and patients grouped according to diabetes status

Preoperative[a]	All patients[b] (n=220)	NDM (n=151)	DMH-NDM (n=34)	DMH-DMH (n=20)	DMT-NDM (n=14)	ANOVA p-value[c]
	mean (95 % CI)	mean (95 % CI)	mean (95 % CI)	mean (95 % CI)	mean (95 % CI)	
Age (years)	44,6 (43,4 - 45,9)	42,1 (40,6 - 43,5)	50,5 (47,7 - 53,3)*	51,5 (48,1 - 55,0)*	48,3 (42,6 - 53,9)	1,4 e^{-8}
Gender (female/male)	150/70	113/38	18/16	9/11	10/4	
height (cm)	171,8 (170,5 - 173,1)	171,0 (169,4 -172,6)	174,5 (171,7 - 177,3)	171,2 (165,8 - 176,5)	174,3 (170,0 - 178,6)	0,195
weight (kg)	125,2 (122,4 - 128,1)	126,3 (122,7 - 129,9)	126,4 (119,2 - 133,6)	117,6 (108,4 - 126,9)	124,1 (114,3 - 133,9)	0,399
BMI (kg/m^2)	42,3 (41,6 - 43,1)	43,1 (42,1 - 44,0)	41,4 (39,5 - 43,3)	40,0 (38,3 - 41,7)	40,9 (37,6 - 44,3)	0,054
Systolic blood pressure (mmHg)	128 (126,0 - 129,9)	126,8 (124,4 - 129,3)	131,2 (126,8 - 135,6)	128,2 (121,3 - 135,1)	131,1 (121,5 - 140,7) (n=13)	0,372
Diastolic blood pressure (mmHg)	82,2 (80,8 - 83,5)	82,0 (80,2 - 83,8)	81,4 (79,0 - 83,7)	82,4 (77,6 - 87,1)	84,8 (81,4 - 88,2) (n=13)	0,773
HbA1c (mmol/mol)	39,0 (37,7 - 40,4)	34,5 (33,9 - 35,1)	48,8 (44,7 - 52,9)	55,2 (50,5 - 59,9)	38,7 (36,5 - 41,0)	5 e^{-12}
Total cholesterol (mmol/L)	4,74 (4,60 - 4,88)	4,98 (4,82 - 5,13)	4,22 (3,84 - 4,61)	4,24 (3,68 - 4,81)	4,21 (3,79 - 4,63)	1,6 e^{-5}
HDL-Cholesterol (mmol/L)	1,15 (1,11 - 1,20)	1,19 (1,15 - 1,24)	1,06 (0,93 - 1,18)	1,09 (0,92 - 1,26)	1,09 (0,96 - 1,21)	0,056
LDL-Cholesterol (mmol/L)	2,88 (2,75 - 3,0)	3,12 (2,98 - 3,26)	2,36 (2,01 - 2,71) (n=32)	2,27 (1,71 - 2,83) (n=18)	2,38 (2,0 - 2,76)	4,4 e^{-7}
VLDL-Cholesterol (mmol/L)	0,71 (0,67 - 0,75)	0,67 (0,62 - 0,72)	0,75 (0,64 - 0,86) (n=32)	0,89 (0,66 - 0,86) (n=18)	0,78 (0,62 - 0,94) (n=13)	0,027
Triglycerides (mmol/L)	1,67 (1,52 - 1,82)	1,50 (1,38 - 1,62)	2,11 (1,43 - 2,79)	2,13 (1,52 - 2,74)	1,66 (1,30 - 2,03)	0,075
**3 months after RYGB**						
weight loss (kg)	22,3 (21,2 - 23,4) (n=168)	22,6 (21,3 - 23,9) (n=116)	21,8 (18,8 - 24,9) (n=26)	23,4 (19,3 - 27,5) (n=14)	20,1 (15,5 - 24,6) (n=11)	0,635
weight loss (%)	17,8 (17,1 - 18,5) (n=168)	17,9 (17,1 - 18,7) (n=116)	17,5 (15,4 - 19,5) (n=26)	19,1 (16,7 - 21,4) (n=14)	16,0 (13,3 - 18,6) (n=11)	0,357
BMI (kg/m^2)	34,7 (34,0 - 35,5) (n=168)	35,2 (34,3 - 36,2) (n=116)	34,3 (32,1 - 36,4) (n=26)	32,4 (30,5 - 34,2) (n=14)	34,3 (32,1 - 36,4) (n=11)	0,056
Systolic blood pressure (mmHg)	131,7 (115,5 - 147,9) (n=151)	133,9 (110,1 - 157,7) (n=103)	128,0 (121,0 - 135,1) (n=24)	124,5 (114,0 - 135,1) (n=13)	124,2 (115,8 - 132,6) (n=10)	0,978
Diastolic blood pressure (mmHg)	76,5 (74,6 - 78,3) (n=151)	76,4 (74,1 - 78,6) (n=103)	76,5 (72,8 - 80,2) (n=24)	75,8 (66,2 - 85,4) (n=13)	75,7 (69,1 - 82,3) (n=10)	0,996
HbA1c (mmol/mol)	34,5 (33,7 - 35,4)	32,1 (31,6 - 32,7)	37,3 (35,4 - 39,1)	48,2 (43,9 - 52,5)	34,6 (33,1 - 36,1)	2,4 e^{-4}
Total cholesterol (mmol/L)	3,92 (3,81 - 4,03)	4,04 (3,91 - 4,16)	3,85 (3,52 - 4,17)	3,30 (3,00 - 3,59)	3,59 (3,17 - 4,01)	4,5 e^{-4}
HDL-Cholesterol (mmol/L)	1,18 (1,14 - 1,21)	1,18 (1,14 - 1,22)	1,14 (1,03 - 1,26)	1,17 (1,00 - 1,34)	1,20 (1,03 - 1,37)	0,885
LDL-Cholesterol (mmol/L)	2,24 (2,14 - 2,34)	2,37 (2,25 - 2,48)	2,15 (1,84 - 2,45)	1,60 (1,34 - 1,85)	1,95 (1,58 - 2,32)	1,6 e^{-5}
VLDL-Cholesterol (mmol/L)	0,51 (0,49 - 0,54)	0,50 (0,47 - 0,52)	0,56 (0,46 - 0,66)	0,54 (0,45 - 0,62)	0,46 (0,37 - 0,56)	0,229
Triglycerides (mmol/L)	1,12 (1,06 - 1,18)	1,08 (1,02 - 1,14)	1,25 (1,03 - 1,48)	1,19 (1,00 - 1,39)	1,02 (0,80 - 1,23)	0,103
**6 months after RYGB**						
weight loss (kg)	30,5 (28,9 - 32,0) (n=152)	31,2 (29,4 - 33,0) (n=104)	29,7 (25,2 - 34,1) (n=23)	28,2 (24,1 - 32,3) (n=15)	28,0 (19,2 - 36,8) (n=10)	0,515
weight loss (%)	24,3 (23,3 - 25,2) (n=152)	24,7 (23,6 - 25,7) (n=104)	24,2 (21,1 - 27,3) (n=23)	23,4 (21,0 - 25,8) (n=15)	22,1 (16,4 - 27,9) (n=10)	0,565
BMI (kg/m^2)	32,1 (31,3 - 32,8) (n=152)	32,6 (31,6 - 33,5) (n=104)	31,1 (28,8 - 33,4) (n=23)	30,4 (28,5 - 32,3) (n=15)	31,7 (30,4 - 33,0) (n=10)	0,183
HbA1c (mmol/mol)	34,5 (33,7 - 35,3) (n=203)	32,3 (31,8 - 32,9) (n=136)	35,8 (34,4 - 37,2) (n=32)	47,0 (43,7 - 50,3)	34,6 (33,0 - 36,1)	1,5 e^{-10}
Total cholesterol (mmol/L)	4,10 (3,96 - 4,24) (n=131)	4,18 (4,01 - 4,35) (n=85)	4,00 (3,61 - 4,38) (n=24)	3,86 (3,38 - 4,33) (n=12)	4,01 (3,49 - 4,54) (n=10)	0,491
HDL-Cholesterol (mmol/L)	1,38 (1,33 - 1,44) (n=131)	1,40 (1,33 - 1,47) (n=85)	1,35 (1,22 - 1,48) (n=24)	1,43 (1,16 - 1,71) (n=12)	1,28 (1,08 - 1,48) (n=10)	0,628
LDL-Cholesterol (mmol/L)	2,25 (2,13 - 2,38) (n=131)	2,35 (2,21 - 2,50) (n=85)	2,13 (1,73 - 2,53) (n=24)	1,86 (1,41 - 2,31) (n=12)	2,18 (1,81 - 2,55) (n=10)	0,124
VLDL-Cholesterol (mmol/L)	0,48 (0,44 -0,51) (n=130)	0,44 (0,41 - 0,47) (n=85)	0,51 (0,43 - 0,59) (n=24)	0,58 (0,44 - 0,71) (n=12)	0,62 (0,34 - 0,91) (n=9)	0,06
Triglycerides (mmol/L)	1,04 (0,97 - 1,11) (n=131)	0,96 (0,89 - 1,02) (n=85)	1,13 (0,95 - 1,30) (n=24)	1,26 (0,96 - 1,56) (n=12)	1,30 (0,68 - 1,91) (n=10)	0,067
**12 months after RYGB**						
weight loss (kg)	35,5 (33,2 - 37,8) (n=115)	36,7 (34,1 - 39,3) (n=82)	32,8 (25,0 - 40,7) (n=14)	31,7 (26,9 - 36,6) (n=14)	34,5 (6,7 - 62,3) (n=5)	0,435
weight loss (%)	29,0 (27,4 - 30,5) (n=115)	30,0 (28,2 - 31,7) (n=82)	26,3 (20,7 - 31,8) (n=14)	26,7 (23,4 - 30,1) (n=14)	26,2 (7,3 - 45,0) (n=5)	0,277
BMI (kg/m^2)	29,8 (28,9 - 30,6) (n=115)	29,6 (28,6 - 30,7) (n=82)	30,6 (27,8 - 33,5) (n=14)	29,0 (26,8 - 31,3) (n=14)	31,5 (28,5 - 34,4) (n=5)	0,676
HbA1c (mmol/mol)	34,7 (33,9 - 35,5) (n=196)	32,6 (32,1 - 33,2) (n=136)	36,1 (34,7 - 37,6) (n=27)	47,1 (44,1 - 50,2) (n=19)	35,1 (34,0 - 36,2) (n=13)	3,3 e^{-11}
Total cholesterol (mmol/L)	4,11 (4,01 - 4,21) (n=190)	4,19 (4,07 - 4,31) (n=131)	4,03 (3,69 - 4,37) (n=27)	3,72 (3,45 - 4,0) (n=19)	4,05 (3,55 - 4,55) (n=12)	0,052
HDL-Cholesterol (mmol/L)	1,52 (1,47 - 1,57) (n=190)	1,54 (1,48 - 1,60) (n=131)	1,48 (1,33 - 1,63) (n=27)	1,48 (1,31 - 1,66) (n=19)	1,47 (1,28 - 1,66) (n=12)	0,704
LDL-Cholesterol (mmol/L)	2,14 (2,06 - 2,23) (n=190)	2,22 (2,12 - 2,31) (n=131)	2,08 (1,81 - 2,35) (n=27)	1,75 (1,52 - 1,98) (n=19)	2,14 (1,70 - 2,59) (n=12)	0,009
VLDL-Cholesterol (mmol/L)	0,45 (0,43 - 0,48) (n=190)	0,43 (0,41 - 0,46) (n=131)	0,47 (0,38 - 0,57) (n=27)	0,51 (0,40 - 0,61) (n=19)	0,44 (0,33 - 0,56) (n=12)	0,478
Triglycerides (mmol/L)	0,99 (0,94 - 1,05) (n=190)	0,96 (0,90 - 1,01) (n=131)	1,05 (0,85 - 1,25) (n=27)	1,09 (0,87 - 1,32) (n=19)	0,98 (0,70 - 1,26) (n=12)	0,543
**24 months after RYGB**						
weight loss (kg)	35,9 (31,1 - 40,7) (n=38)	38,4 (32,4 - 44,4) (n=26)	30,1 (18,2 - 42,0) (n=7)	36,3 (24 - 48,6) (n=2)	27,5 (16 - 46,8) (n=3)	0,436
weight loss (%)	29,0 (25,7 - 32,3) (n=38)	31,3 (27,5 - 35,0) (n=26)	23,5 (13,8 - 33,1) (n=7)	33,3 (28,3 - 38,3) (n=2)	19,8 (12,2 - 32,2) (n=3)	0,088
BMI (kg/m^2)	29,7 (28,3 - 31,2) (n=38)	28,8 (27,1 - 30,5) (n=26)	32,6 (28,2 - 36,9) (n=7)	26,8 (25,9 - 27,7) (n=2)	33,1 (28,5 - 36,4) (n=3)	0,087
HbA1c (mmol/mol)	36,0 (35,0 - 36,9) (n=197)	33,3 (32,8 - 33,9) (n=134)	37,5 (35,7 - 39,3) (n=28)	51,1 (47,5 - 54,6)	35,9 (34,6 - 37,2) (n=14)	6,2 e^{-12}
Total cholesterol (mmol/L)	4,20 (4,07 - 4,32) (n=187)	4,27 (4,12 - 4,42) (n=128)	4,29 (3,91 - 4,66) (n=26)	3,50 (3,24 - 3,75) (n=19)	4,26 (3,77 - 4,76) (n=13)	0,002
HDL-Cholesterol (mmol/L)	1,57 (1,52 - 1,63) (n=187)	1,61 (1,54 - 1,67) (n=128)	1,56 (1,39 - 1,72) (n=26)	1,43 (1,26 - 1,59) (n=19)	1,50 (1,27 - 1,73) (n=13)	0,223
LDL-Cholesterol (mmol/L)	2,16 (2,06 - 2,26) (n=187)	2,23 (2,11 - 2,35) (n=128)	2,20 (1,86 - 2,53) (n=26)	1,59 (1,37 - 1,81) (n=19)	2,26 (1,92 - 2,60) (n=13)	0,003
VLDL-Cholesterol (mmol/L)	0,46(0,43 - 0,49) (n=186)	0,44 (0,40 - 0,47) (n=127)	0,53 (0,41 - 0,66) (n=26)	0,48 (0,37 - 0,59) (n=19)	0,50 (0,36 - 0,64) (n=13)	0,327
Triglycerides (mmol/L)	1,03 (0,96 - 1,10) (n=187)	0,97 (0,90 - 1,04) (n=128)	1,19 (0,92 - 1,46) (n=26)	1,06 (0,82 - 1,30) (n=19)	1,12 (0,82 - 1,43) (n=13)	0,322

Values are shown as mean with a 95 % confidence interval of the mean, except when n < 3, where it is shown as mean with minimum and maximum values.
RYGB, Roux-en-y gastric bypass surgery; HbA1c, Acylated hemogobin 1c; HDL, High-density lipoprotein; LDL, Low-density lipoprotein; VLDL, Very low-density lipoprotein;
NDM, patients without diabetes mellitus (DM); DMH-NDM, patients with DM (criteria for diagnosis confirmed in avaliable lab data) in remission after RYGB; DMH-DMH, patients with DM not in remission after RYGB surgery; DMT-NDM, patients in treatment for possible DM (criteria for diagnisis could not be confirmed in avaliable lab data) in remission after RYGB;
[a]Age is on the day of surgery, preoperative clinical data represent the closest avaliable before surgery.
[b]All patients also include one patient with DM in biochemical remission after RYGB surgery, who continued treatment with antidiabetic medicine.
[c] P-value for comaparison of subgroup is from one way analysis of variance (ANOVA), and does for aparameters with inequality of variances include a Welch-Sattertwaithe correction.
All clinical data was not recorded for all patients at all time points. If the number of patients with clinical data at any time point is less than 95 % of the group total, the number is specified.
Data for systolic and diastolic blood pressure were for a majority of patients not available later than 3 months after surgery.
