# Supplementary material for: Changes in Serum Sphingomyelin After Roux-en-Y Gastric Bypass Surgery Are Related to Diabetes Status
Source: Front Endocrinol (Lausanne). 2018 Apr 25;9:172. doi: 10.3389/fendo.2018.00172 (PMC5996901; doi:10.3389/fendo.2018.00172)
Supplement: Supplementary file 2 [file table_2.PDF]

Supplementary table S2. Serum sphingomyelin concentration and its relation to body weight and body mass before and after RYGB-surgery across non-diabetic and diabetic patient subgroups.

		before RYGB<br>mean (95 % CI)	3 months after RYGB<br>mean (95 % CI)	6 months after RYGB<br>mean (95 % CI)	12 months after RYGB<br>mean (95 % CI)	24 months after RYGB<br>mean (95 % CI)
**All patients**						
Number (N)	All (f/m)	220 (150/70)	220 (150/70)	158 (114/44)	148 (103/45)	89 (64/25)
SM (µmol/L)	All	413,1 (400,7 - 425,5)	394,0 (382,2 - 405,8)****	391,7 (378,4 - 405,1)********	389,4 (378,5 - 400,3)*******	376,5 (361,5 - 391,6)****
	Female	434,3 (419,8 - 448,9)	416,3 (402,0 - 430,6)**	410,4 (394,4 - 426,4)*****	412,2 (400,7 - 423,6)*****	394,4 (378,5 - 410,3)*****
	Male	367,6 (347,8 - 387,4)	346,3 (330,1 - 362,5)*	343,3 (325,2 - 361,4)**	337,3 (320,3 - 354,3)**	330,8 (301,6 - 360,1)
SM per kg bodyweight (µmol/(L*kg))	All	3,40 (3,27 - 3,53)	4,02 (3,84 - 4,20) (n=168)**********	4,34 (4,11 - 4,58) (n=118)**********	4,57 (4,28 - 4,85) (n=92)**********	4,45 (3,88 - 5,02) (n=26)*****
	Female	3,75 (3,60 - 3,89)	4,40 (4,19 - 4,60) (n=118)**********	4,75 (4,49 - 5,01) (n=87)**********	5,14 (4,84 - 5,44) (n=63)**********	4,88 (4,25 - 5,51) (n=19)****
	Male	2,66 (2,50 - 2,83)	3,12 (2,91 - 3,33) (n=50)********	3,20 (2,96 - 3,44) (n=31)***	3,32 (3,02 - 3,62) (n=29)*****	3,29 (2,42 - 4,16) (n=7)*
SM per kg/m2 BMI ((µmol*m2)/(L*kg))	All	9,90 (9,57 - 10,23)	11,67 (11,22 - 12,12) (n=168)**********	12,50 (11,92 - 13,08) (n=118)**********	13,16 (12,48 - 13,83) (n=92)**********	12,82 (11,43 - 14,21) (n=26)*****
	Female	10,45 (10,06 - 10,83)	12,29 (11,75 - 12,83) (n=118)**********	13,22 (12,55 - 13,89) (n=87)**********	14,29 (13,55 - 15,03) (n=63)**********	13,64 (12,04 - 15,25) (n=19)****
	Male	8,73 (8,21 - 9,25)	10,21 (9,53 - 10,89) (n=50)********	10,46 (9,59 - 11,34) (n=31)***	10,69 (9,71 - 11,67) (n=29)*****	10,58 (7,93 - 13,24) (n=7)*
**NDM**						
Number (N)	All (f/m)	151 (113/38)	151 (113/38)	113 (86/27)	101 (78/23)	62 (50/12)
SM (µmol/L)	All	437,2 (423,7 - 450,7)	411,0 (397,3 - 424,7)*****	403 (387,3 - 418,6)*********	401,9 (389,3-414,5)*********	388,3 (371,0 - 405,7)******
	Female	449,1 (433,5 - 464,7)	431,0 (415,2 - 446,8)**	421,1 (403,3 - 438,9)******	420,6 (408,3 - 432,8)*****	398,2 (379,4 - 416,9)*****
	Male	401,7 (376,9 - 426,4)	351,5 (333,5 - 369,4)****	345,2 (322,1 - 368,2)***	338,6 (315,7 - 361,5)****	347,3 (306,0 - 388,6)*
SM per kg bodyweight (µmol/(L*kg))	All	3,58 (3,43 - 3,74)	4,19 (3,96 - 4,42) (n=116)**********	4,45 (4,15 - 4,75) (n=84)**********	4,81 (4,46 - 5,16) (n=66)**********	4,82 (4,17 - 5,46) (n=18)****
	Female	3,85 (3,68 - 4,02)	4,54 (4,29 - 4,78) (n=89)**********	4,86 (4,55 - 5,18) (n=64)**********	5,31 (4,97 - 5,66) (n=50)**********	5,01 (4,28 - 5,75) (n=15)***
	Male	2,78 (2,57 - 3,0)	3,05 (2,79 - 3,30) (n=27)***	3,13 (2,81 - 3,45) (n=20)*	3,26 (2,88 - 3,63) (n=16)**	3,82 (2,65 - 4,99) (n=3)
SM per kg/m2 BMI ((µmol*m2)/(L*kg))	All	10,34 (9,95 - 10,72)	12,03 (11,47 - 12,60) (n=116)**********	12,70 (11,96 - 13,43) (n=84)**********	13,69 (12,86 - 14,51) (n=66)**********	13,81 (12,14 - 15,47) (n=18)****
	Female	10,72 (10,28 - 11,16)	12,65 (12,01 - 13,29) (n=89)**********	13,47 (12,64 - 14,29) (n=64)**********	14,71 (13,86 - 15,56) (n=50)**********	14,08 (12,09 - 16,07) (n=15)***
	Male	9,21 (8,51 - 9,91)	10,01 (9,10 - 10,91) (n=27)***	10,22 (9,10 - 11,35) (n=20)	10,49 (9,23 - 11,74) (n=16)**	12,46 (9,13 - 15,80) (n=3)
**DMH-NDM**						
Number (N)	All (f/m)	34 (18/16)	34 (18/16)	21 (14/7)	19 (11/8)	14 (8/6)
SM (µmol/L)	All	360,3 (325,0 - 395,6)	373,5 (340,4 - 406,7)	370,1 (328,0 - 412,2)	371,6 (336,2 - 407,1)	356,6 (306,6 - 406,7)
	Female	384,9 (332,4 - 437,4)	385,9 (337,2 - 434,6)	378,5 (321,4 - 435,6)	394,7 (356,7 - 432,7)	377,8 (324,1 - 431,4)
	Male	332,6 (283,9 - 381,4)	359,6 (310,2 - 409,0)	353,3 (276,7 - 429,9)	339,9 (268,3 - 411,5)	328,5 (213,1 - 443,9)
SM per kg bodyweight (µmol/(L*kg))	All	2,91 (2,59 - 3,22)	3,66 (3,29 - 4,03) (n=26)*****	4,13 (3,68 - 4,58) (n=15)***	4,08 (3,31 - 4,85) (n=10)**	3,15 (1,41 - 4,90) (n=4)
	Female	3,30 (2,85 - 3,74)	3,98 (3,46 - 4,49) (n=16)**	4,26 (3,74 - 4,78) (n=12)**	4,57 (3,49 - 5,65) (n=6)*	3,96 (3,87 - 4,04) (n=2)
	Male	2,47 (2,10 - 2,83)	3,16 (2,74 - 3,57) (n=10)**	3,61 (1,85 - 5,37) (n=3)	3,35 (2,15 - 4,55) (n=4)*	2,35 (1,64 - 3,06) (n=2)
SM per kg/m2 BMI ((µmol*m2)/(L*kg))	All	8,79 (7,91 - 9,67)	10,97 (9,97 - 11,96) (n=26)*****	12,07 (10,72 - 13,42) (n=15)***	12,11 (10,38 - 13,85) (n=10)**	9,79 (4,93 - 14,65) (n=4)
	Female	9,41 (8,10 - 10,73)	11,35 (9,92 - 12,77) (n=16)**	12,23 (10,70 - 13,75) (n=12)**	13,07 (10,75 - 15,38) (n=6)*	11,90 (11,68 - 12,12) (n=2)
	Male	8,09 (6,90 - 9,28)	10,36 (8,87 - 11,84) (n=10)**	11,45 (3,98 - 18,92) (n=3)	10,69 (7,16 - 14,22) (n=4)*	7,67 (5,44 - 9,90) (n=2)
**DMH-DMH**						
Number (N)	All (f/m)	20 (9/11)	20 (9/11)	12 (5/7)	15 (5/10)	7 (2/5)
SM (µmol/L)	All	343,2 (303,8 - 382,5)	319,1 (293,3 - 344,8)	340,3 (296,2 - 384,5)	328,7 (300,6 - 356,9)	327,6 (284,7 - 370,5)
	Female	381,6 (300,9 - 462,3)	339,1 (297,6-380,6)	369,4 (250,6 - 488,2)	343,4 (262,2 - 424,6)	379,0 (379,0 - 379,0)
	Male	311,7 (280,4 - 343,1)	302,6 (267,4 - 337,9)	319,6 (283,4 - 355,8)	321,4 (289,8 - 353,0)	307,0 (260,9 - 353,1)
SM per kg bodyweight (µmol/(L*kg))	All	3,03 (2,55 - 3,51)	3,39 (2,83 - 3,95) (n=14)**	3,91 (2,96 - 4,87) (n=11)**	3,73 (3,0 - 4,45) (n=11)***	5,22 (4,20 - 6,24) (n=2)
	Female	3,75 (2,95 - 4,55)	4,04 (2,96 - 5,11) (n=5)	4,79 (2,74 - 6,84)	4,54 (3,06 - 6,02) (n=3)**	6,24 (n=1)
	Male	2,44 (2,09 - 2,80)	3,03 (2,37 - 3,69) (n=9)**	3,18 (2,48 - 3,89) (n=6)*	3,42 (2,51 - 4,33) (n=8)**	4,20 (n=1)
SM per kg/m2 BMI ((µmol*m2)/(L*kg))	All	8,64 (7,62 - 9,66)	10,02 (8,78 - 11,27) (n=14)**	11,38 (9,57 - 13,18) (n=11)**	11,40 (9,30 - 13,50) (n=11)***	13,19 (12,71 - 13,68) (n=2)
	Female	9,82 (7,90 - 11,74)	10,59 (8,53 - 12,65) (n=5)	12,41 (8,93 - 15,89)*	12,41 (7,89 - 16,93) (n=3)**	13,68 (n=1)
	Male	7,68 (6,80 - 8,56)	9,71 (7,85 - 11,56) (n=9)**	10,52 (7,90 - 13,13) (n=6)*	11,02 (8,08 - 13,96) (n=8)**	12,71 (n=1)
**between groups ANOVA p-value**						
SM	Female	0,003	0,002	0,119	0,006	0,673
	Male	0,001	0,037	0,531	0,715	0,598
SM per kg bodyweight	Female	0,061	0,142	0,309	0,222	0,347
	Male	0,139	0,893	0,531	0,897	0,164
SM per kg/m2 BMI	Female	0,076	0,108	0,392	0,205	0,712
	Male	0,045	0,823	0,723	0,904	0,166

Data are reported as mean with a 95 % confidence interval (CI) of the mean except where n = 1 (no CI is reported) and n = 2 (CI is replaced with minimum and maximum values).
Weight and/or BMI where not registred for all patients at all time points. The actual number of patients with available data is specified where it was less than 95 % of the total of patients in the group at that particular timepoint.
RYGB, Roux-en-y gastric bypass; SM, Serum sphingomyelin concentration; BMI, body mass index; f, female; m, male; NDM, patients without diabetes mellitus (DM); DMH-NDM, patients with DM in remission after RYGB;
DMH-DMH, patients with DM not in remission after RYGB. * significantly different from corresponding value before surgery;
* p<0,05 ** p<0,005 *** p<5e-4 **** p<5e-5 ***** p<5e-6 ****** p<5e-7 ******* p<5e-8 ******** p<5e-9 ********* p<5e-10 ********** p-values between 5e-11 and 5e-25.
Differences between subgroups is shown with ANOVA p-values, where values < 0,05 are considered significant.
