# Supplementary material for: Changes in Serum Sphingomyelin After Roux-en-Y Gastric Bypass Surgery Are Related to Diabetes Status
Source: Front Endocrinol (Lausanne). 2018 Apr 25;9:172. doi: 10.3389/fendo.2018.00172 (PMC5996901; doi:10.3389/fendo.2018.00172)
Supplement: Supplementary file 3 [file table_3.PDF]

Supplementary Table S3. Correlations between serum sphingomyelin and routine lipids before surgery and 3, 6, 12, and 24 months after surgery.

Correlations

Before surgery			SM	total cholesterol	HDL-cholesterol	LDL-cholesterol	VLDL-cholesterol	Triglycerides
Spearman's rho	SM	Correlation Coefficient	1,000	,722**	,400**	,707**	-,110	-,108
		Sig. (2-tailed)		,000	,000	,000	,112	,115
		N	220	214	214	210	209	214
	total cholesterol	Correlation Coefficient	,722**	1,000	,266**	,929**	,219**	,217**
		Sig. (2-tailed)	,000		,000	,000	,001	,001
		N	214	214	214	210	209	214
	HDL-cholesterol	Correlation Coefficient	,400**	,266**	1,000	,102	-,319**	-,351**
		Sig. (2-tailed)	,000	,000		,141	,000	,000
		N	214	214	214	210	209	214
	LDL-cholesterol	Correlation Coefficient	,707**	,929**	,102	1,000	,034	,041
		Sig. (2-tailed)	,000	,000	,141		,621	,554
		N	210	210	210	210	209	210
	VLDL-cholesterol	Correlation Coefficient	-,110	,219**	-,319**	,034	1,000	,992**
		Sig. (2-tailed)	,112	,001	,000	,621		,000
		N	209	209	209	209	209	209
	Triglycerides	Correlation Coefficient	-,108	,217**	-,351**	,041	,992**	1,000
		Sig. (2-tailed)	,115	,001	,000	,554	,000	
		N	214	214	214	210	209	214

**. Correlation is significant at the 0.01 level (2-tailed).

3 months after RYGB			SM	total cholesterol	HDL-cholesterol	LDL-cholesterol	VLDL-cholesterol	Triglycerides
Spearman's rho	SM	Correlation Coefficient	1,000	,801**	,350**	,731**	,138*	,117
		Sig. (2-tailed)		,000	,000	,000	,041	,084
		N	220	220	220	220	220	220
	total cholesterol	Correlation Coefficient	,801**	1,000	,229**	,929**	,348**	,328**
		Sig. (2-tailed)	,000		,001	,000	,000	,000
		N	220	220	220	220	220	220
	HDL-cholesterol	Correlation Coefficient	,350**	,229**	1,000	-,030	-,229**	-,254**
		Sig. (2-tailed)	,000	,001		,662	,001	,000
		N	220	220	220	220	220	220
	LDL-cholesterol	Correlation Coefficient	,731**	,929**	-,030	1,000	,242**	,232**
		Sig. (2-tailed)	,000	,000	,662		,000	,001
		N	220	220	220	220	220	220
	VLDL-cholesterol	Correlation Coefficient	,138*	,348**	-,229**	,242**	1,000	,981**
		Sig. (2-tailed)	,041	,000	,001	,000		,000
		N	220	220	220	220	220	220
	Triglycerides	Correlation Coefficient	,117	,328**	-,254**	,232**	,981**	1,000
		Sig. (2-tailed)	,084	,000	,000	,001	,000	
		N	220	220	220	220	220	220

**. Correlation is significant at the 0.01 level (2-tailed).

*. Correlation is significant at the 0.05 level (2-tailed).

6 months after RYGB			SM	total cholesterol	HDL-cholesterol	LDL-cholesterol	VLDL-cholesterol	Triglycerides
Spearman's rho	SM	Correlation Coefficient	1,000	,784**	,443**	,678**	-,047	-,036
		Sig. (2-tailed)		,000	,000	,000	,647	,724
		N	158	97	97	97	97	97
	total cholesterol	Correlation Coefficient	,784**	1,000	,309**	,890**	,148	,180*
		Sig. (2-tailed)	,000		,000	,000	,093	,039
		N	97	131	131	131	130	131
	HDL-cholesterol	Correlation Coefficient	,443**	,309**	1,000	-,028	-,332**	-,308**
		Sig. (2-tailed)	,000	,000		,753	,000	,000
		N	97	131	131	131	130	131
	LDL-cholesterol	Correlation Coefficient	,678**	,890**	-,028	1,000	,093	,117
		Sig. (2-tailed)	,000	,000	,753		,290	,185
		N	97	131	131	131	130	131
	VLDL-cholesterol	Correlation Coefficient	-,047	,148	-,332**	,093	1,000	,974**
		Sig. (2-tailed)	,647	,093	,000	,290		,000
		N	97	130	130	130	130	130
	Triglycerides	Correlation Coefficient	-,036	,180*	-,308**	,117	,974**	1,000
		Sig. (2-tailed)	,724	,039	,000	,185	,000	
		N	97	131	131	131	130	131

**. Correlation is significant at the 0.01 level (2-tailed).

*. Correlation is significant at the 0.05 level (2-tailed).

12 months after RYGB			SM	total cholesterol	HDL-cholesterol	LDL-cholesterol	VLDL-cholesterol	Triglycerides
Spearman's rho	SM	Correlation Coefficient	1,000	,716**	,518**	,527**	,059	,058
		Sig. (2-tailed)		,000	,000	,000	,490	,503
		N	148	138	138	138	138	138
	total cholesterol	Correlation Coefficient	,716**	1,000	,398**	,868**	,387**	,388**
		Sig. (2-tailed)	,000		,000	,000	,000	,000
		N	138	190	190	190	190	190
	HDL-cholesterol	Correlation Coefficient	,518**	,398**	1,000	,000	-,239**	-,247**
		Sig. (2-tailed)	,000	,000		,996	,001	,001
		N	138	190	190	190	190	190
	LDL-cholesterol	Correlation Coefficient	,527**	,868**	,000	1,000	,335**	,340**
		Sig. (2-tailed)	,000	,000	,996		,000	,000
		N	138	190	190	190	190	190
	VLDL-cholesterol	Correlation Coefficient	,059	,387**	-,239**	,335**	1,000	,974**
		Sig. (2-tailed)	,490	,000	,001	,000		,000
		N	138	190	190	190	190	190
	Triglycerides	Correlation Coefficient	,058	,388**	-,247**	,340**	,974**	1,000
		Sig. (2-tailed)	,503	,000	,001	,000	,000	
		N	138	190	190	190	190	190

**. Correlation is significant at the 0.01 level (2-tailed).

24 months after RYGB			SM	total cholesterol	HDL-cholesterol	LDL-cholesterol	VLDL-cholesterol	Triglycerides
Spearman's rho	SM	Correlation Coefficient	1,000	,698**	,418**	,550**	-,098	-,077
		Sig. (2-tailed)		,000	,000	,000	,362	,474
		N	89	88	88	88	88	88
	total cholesterol	Correlation Coefficient	,698**	1,000	,310**	,903**	,294**	,307**
		Sig. (2-tailed)	,000		,000	,000	,000	,000
		N	88	187	187	187	186	187
	HDL-cholesterol	Correlation Coefficient	,418**	,310**	1,000	-,016	-,344**	-,358**
		Sig. (2-tailed)	,000	,000		,828	,000	,000
		N	88	187	187	187	186	187
	LDL-cholesterol	Correlation Coefficient	,550**	,903**	-,016	1,000	,254**	,282**
		Sig. (2-tailed)	,000	,000	,828		,000	,000
		N	88	187	187	187	186	187
	VLDL-cholesterol	Correlation Coefficient	-,098	,294**	-,344**	,254**	1,000	,977**
		Sig. (2-tailed)	,362	,000	,000	,000		,000
		N	88	186	186	186	186	186
	Triglycerides	Correlation Coefficient	-,077	,307**	-,358**	,282**	,977**	1,000
		Sig. (2-tailed)	,474	,000	,000	,000	,000	
		N	88	187	187	187	186	187

**. Correlation is significant at the 0.01 level (2-tailed).

RYGB, Roux-en-y gastric bypass surgery; SM, Serum sphingomyelin; HDL, High-density lipoprotein; LDL, Low-density lipoprotein; VLDL, Very low-density lipoprotein
